# Solvent‐Activated Hafnium‐Containing Zeolites Enable Selective and Continuous Glucose–Fructose Isomerisation

**DOI:** 10.1002/anie.202006718

**Published:** 2020-08-31

**Authors:** Luca Botti, Simon A. Kondrat, Ricardo Navar, Daniele Padovan, Juan S. Martinez‐Espin, Sebastian Meier, Ceri Hammond

**Affiliations:** ^1^ Department of Chemical Engineering Imperial College London London SW7 2AZ UK; ^2^ Cardiff Catalysis Institute Cardiff University Cardiff CF10 3AT UK; ^3^ Department of Chemistry Loughborough University Loughborough UK; ^4^ Haldor Topsøe A/S Haldor Topsøes Allé 1 2800-Kgs. Lyngby Denmark; ^5^ Department of Chemistry Technical University of Denmark Kemitorvet Building 207 2800-Kgs. Lyngby Denmark

**Keywords:** biomass, heterogeneous catalysis, glucose, hafnium, zeolites

## Abstract

The isomerisation of glucose to fructose is a critical step towards manufacturing petroleum‐free chemicals from lignocellulosic biomass. Herein we show that Hf‐containing zeolites are unique catalysts for this reaction, enabling true thermodynamic equilibrium to be achieved in a single step during intensified continuous operation, which no chemical or biological catalyst has yet been able to achieve. Unprecedented single‐pass yields of 58 % are observed at a fructose selectivity of 94 %, and continuous operation for over 100 hours is demonstrated. The unexpected performance of the catalyst is realised following a period of activation within the reactor, during which time interaction with the solvent generates a state of activity that is absent in the synthesised catalyst. Mechanistic studies by X‐ray absorption spectroscopy, chemisorption FTIR, *operando* UV/Vis and ^1^H–^13^C HSQC NMR spectroscopy indicate that activity arises from isolated Hf^IV^ atoms with monofunctional acidic properties.

## Graphical abstract

Hafnium zeolites are shown to catalyse glucose–fructose isomerisation to thermodynamic equilibrium during continuous operation, which no chemical catalyst has yet been able to achieve. Fructose yields of 58 % are observed at a selectivity of 94 %. Structure–activity relationships are developed with X‐ray absorption spectroscopy, chemisorption FTIR, operando UV/Vis and HSQC NMR spectroscopy.

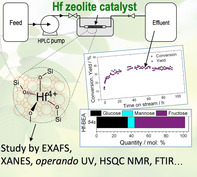

## Introduction

Pressing environmental and societal concerns are driving researchers to develop new processes with minimised reliance on fossil resources.^[1]^ In this context, sustainable chemical manufacture is a major challenge, since over 95 % of the organic chemicals currently employed in the chemical industry are sourced from coal, oil and natural gas.^[2]^ Amongst renewable resources, lignocellulosic biomass is especially promising, given its abundance, high chemical functionality and geographic diversity. Yet, its use as a chemical feedstock requires the development of selective catalytic technologies that are able to transform this valuable feedstock into useful end products in an economically and environmentally efficient manner.

The isomerisation of glucose to fructose (GI) is a key step towards converting glucose (the most abundant fraction of lignocellulose) into a variety of commercially relevant chemicals, including furanics, levulinates and unsaturated hydroxyesters.^[3]^ Although enzymes catalyse GI to single pass fructose yields of up to 42 %,^[4]^ development of a solid (heterogeneous) catalyst capable of performing this reaction would be an important step forward, both by improving operational flexibility (temperature, pH, feed purity)^[5]^ and facilitating process intensification.^[6]^ To date, it has become generally appreciated that tin (Sn) containing zeolites (particularly Sn‐BEA) possess the greatest potential for chemo‐catalytic GI.^[7]^ Zeolites are crystalline, microporous silicates, which in the case of Sn‐BEA is a three‐dimensional silicate containing dilute amounts of Lewis acidic Sn^IV^ atoms within its lattice.^[8]^ However, although the high activity of Sn‐BEA for GI has received widespread attention, several negative aspects of its performance are less appreciated, such as its poor stability in the polar solvents required for biomass conversion,^[9]^ and its tendency to catalyse a variety of competitive and/or degradation reactions at operational conditions.^[10]^ The latter is especially problematic, as it wastes a precious resource^[1b]^ and negatively impacts process economics. However, no better alternatives to Sn‐BEA have yet been found. Consequently, heterogeneous catalysis is unable to reach the levels of performance exhibited by enzymes, and renewable chemical production by scalable chemo‐catalytic methods remains limited by the enduring bottleneck of selective glucose isomerisation.

Here we show that hafnium (Hf)‐containing zeolites are uniquely able to perform GI at unprecedented levels of selectivity, even at commercially relevant operational conditions. The optimal catalyst (Hf‐BEA, Hf/Si molar ratio=202, prepared by hydrothermal synthesis^[11]^) achieves single pass fructose yields of 58 %, at a fructose selectivity of 94 %, even during continuous operation for over 100 h on stream. Its unique performance is maintained even at elevated temperature (<140 °C), which is an advantage over biological catalysts that are only active at low temperature, where thermodynamics favours the reactant side of equilibrium, and reactor productivity is limited.^[7b]^ Structure‐activity relationships indicate that activity arises from isolated Hf^IV^ atoms with monofunctional acidic properties, which permit high productivity isomerisation to be achieved without the undesirable side reactions that plague existing chemo‐catalysts.

## Results and Discussion

**Kinetic studies**. Our preliminary experiments revealed that amongst a range of analogous Lewis acidic silicate zeolites—each of which was prepared by fluoride‐media hydrothermal synthesis (SI Figure S1)—Sn‐BEA was the best performing catalyst for GI during batch operation in methanol (MeOH), which was chosen as solvent as it maximises catalyst stability and facilitates downstream processing (Table 1).[^9a^, ^9b^] Other Lewis acidic silicates were either much less active (Ti‐BEA, Zr‐BEA), or inactive (Hf‐BEA), for GI. At the low levels of substrate conversion achieved during batch operation (*X*_Glu_<20 %), Sn‐BEA was also relatively selective to fructose (*S*_Fru_<68 %), and was somewhat more selective than Zr‐ and Ti‐BEA (45 and 56 %, respectively).

**Table 1:** Physical, chemical, and reactivity data for various Lewis acidic silicates explored for glucose isomerisation in batch mode.^[a]^

Entry	Catalyst	SSA [m^2^ g^−1^]	*V*_micro_ [cm^3^ g^−1^]	Si/M	TOF for GI [h^−1^]	*S*_Fru_ at *X*_Glu_=20 % (%)
1	Sn‐BEA	398	0.21	198	95	68
2	Zr‐BEA	414	0.21	189	16	45
3	Ti‐BEA	390	0.20	204	15	56
4	Hf‐BEA	404	0.22	202	0	–

[a] Porosity data determined from N_2_ isotherms. Si/M molar ratio determined by ICP‐MS. Turnover frequency (TOF) values calculated from batch GI experiments at *t*=2 minutes. Reaction conditions: 1 wt % glucose in MeOH, 4 g reaction solution, glucose/metal ratio of 50, 110 °C, autogenic pressure.

**Figure 1:**
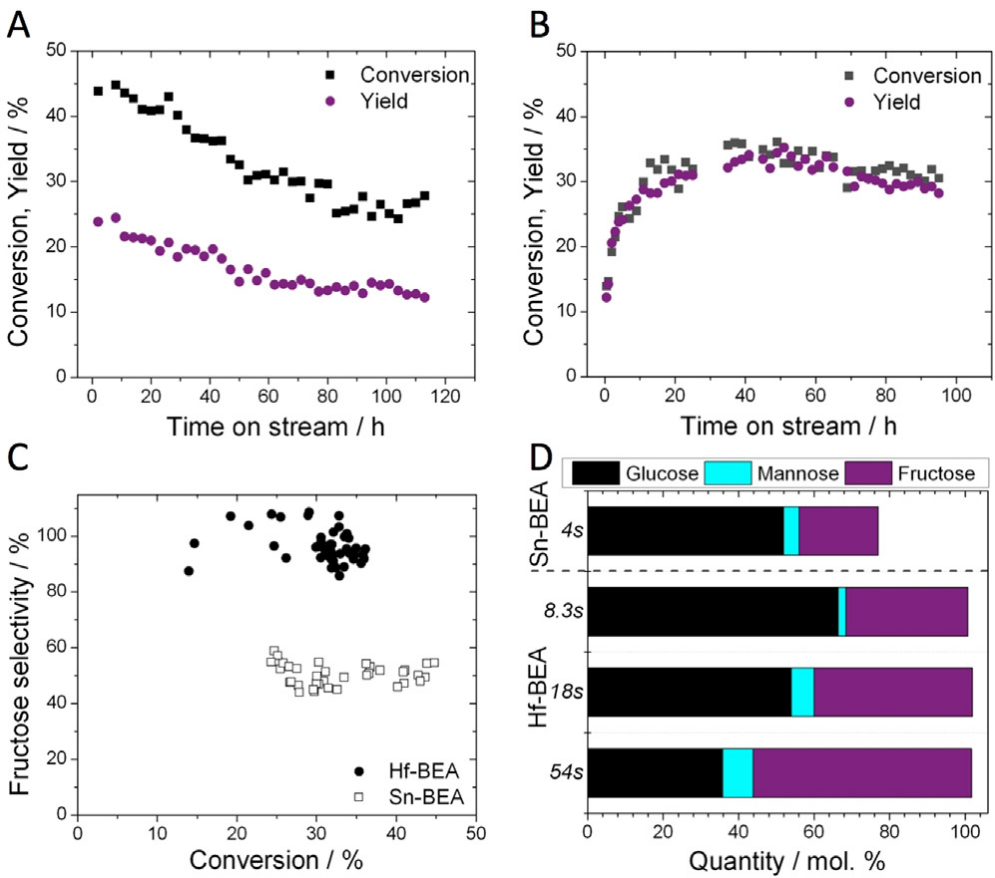
Kinetic data for the isomerisation of glucose to fructose over Sn‐ and Hf‐containing BEA zeolites. A) Time on stream data for Sn‐BEA; B) Time on stream data for Hf‐BEA; C) Glucose conversion versus fructose selectivity profile for Sn‐BEA and Hf‐BEA; D) Product distribution achieved with Sn‐BEA and Hf‐BEA at various contact times between 4 and 54 seconds. General reaction conditions: 1 wt % glucose in methanol, 110 °C. Precise conditions including WHSV of each experiment are provided in the Supporting Information Table S1.

However, major differences in performance for the Lewis acidic silicates described in Table 1 were observed when GI was performed at continuous processing conditions. In continuous processes, reactants are continuously pumped over a suspended solid catalyst bed, and the reaction occurs during the time the solution resides on the catalyst within the reactor (“contact time”). Such reactors are widely employed in the base chemical industry, due to their increased levels of productivity, safety and scalability.^[12]^ Figure 1 A‐D presents the time on stream data for two particular Lewis acidic silicates, Sn‐BEA and Hf‐BEA, for continuous GI at 110 °C, alongside their conversion vs. selectivity profiles and product distribution profiles. As can be seen (Figure 1 A), Sn‐BEA exhibited an initially high level of substrate conversion (45 %) at the conditions employed (SI Table S1, Entry 2). However, relatively rapid deactivation occurred, resulting in more than 40 % loss of activity over the course of 113 h on stream. A single deactivation regime was determined, characterized by a deactivation rate constant of 0.00715 X% h^−1^ (SI Figure S2).^[13]^ In contrast, Hf‐BEA exhibited a very different kinetic profile (Figure 1 B). After an initial induction period lasting several hours, Hf‐BEA reached similar levels of activity to Sn‐BEA. Its performance was maintained over the following 72 h on stream, with conversion and yield fluctuating by the typical experimental error of the testing and analytical protocols (5–8 %). After the induction period, a deactivation constant of 0.00281 X% h^−1^ was determined, demonstrating Hf‐BEA to be more stable than Sn‐BEA (SI Figure S3).

In addition, analysis of the product distribution revealed Hf‐BEA to be substantially more selective to fructose than Sn‐BEA. When comparing the fructose selectivity at all levels of conversion (Figure 1 C), Hf‐BEA was approximately twice as selective as Sn‐BEA, converting glucose solely to fructose and mannose, the two thermodynamic products expected from GI, at a carbon balance level of 100 % (Figure 1 D). In contrast, only around half of the glucose converted by Sn‐BEA became fructose, and a substantial (>20 %) loss of carbon balance was observed (Figure 1 D), indicating that unwanted by‐products were formed. Notably, the exceptional selectivity of Hf‐BEA was maintained even at extended contact times (Figure 1 D). As can be seen, increasing the contact time from 8 seconds to 54 seconds increased the quantity of glucose converted, from 33.6 % to 66.2 %. Yet, despite operating at extremely high conversion, the excellent selectivity of Hf‐BEA remained, resulting in an unprecedented single‐pass fructose yield of 57.9 % being achieved. Notably, no loss of carbon balance was observed in this run, strongly indicating that competitive and/or consecutive side reactions remained absent even at high levels of conversion. In fact, the product distribution achieved at these conditions is representative of the thermodynamic equilibrium mixture expected at 110 °C, representing the first time chemo‐catalysis has been able to achieve this feat for GI.^[14]^

We note here that the induction period observed with Hf‐BEA (Figure 1 B) could be avoided by activating the catalyst in MeOH at 110 °C for 20 h prior to introducing glucose into the feed, which ensured that no valuable substrate was wasted during operation (SI Figure S4). Furthermore, although Zr‐BEA also exhibited an induction period and reached high levels of selectivity (SI Figure S5), its absolute activity was 5 times lower than that of Hf‐BEA, and its selectivity was also found to be approximately 20 % lower, at otherwise identical conditions.

**Figure 2:**
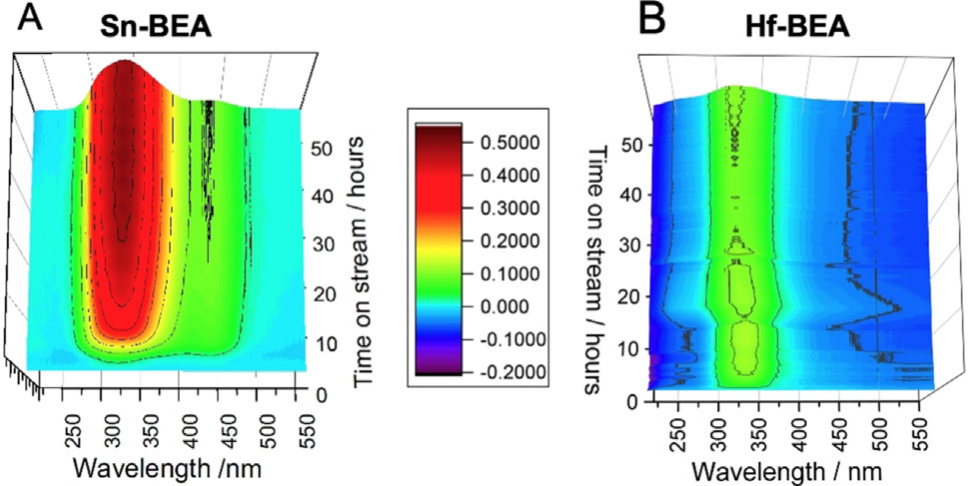
*Operando* UV/Vis measurements for A) Sn‐BEA and B) Hf‐BEA catalysed GI at 110 °C, permitting the absorption spectra of the system to be measured continuously alongside the kinetics of the system. General reaction conditions: 1 wt % glucose in methanol, 110 °C.

To understand the nature of the overall reaction network, and hence better understand the origin of the improved performance of Hf‐BEA relative to Sn‐BEA, detailed studies of both product mixtures were undertaken. Firstly, *operando* UV/Vis was performed during continuous GI over Sn‐BEA and Hf‐BEA, according to our recently benchmarked procedure.^[15]^ We note that the kinetic data obtained with the *operando* reactor matched that obtained in the conventional flow reactor (SI Figure S6),^[15]^ and that both experiments were tailored so that similar levels of substrate conversion were attained during both reactions. The resulting spectra of the first 50 h on stream under the UV probe are shown in Figure 2 A,B. Spectra are presented in difference mode, so that optical features formed during the reaction exhibit positive signals, whereas optical features decreasing during the reaction exhibit negative signals. Both experiments exhibited a strong, positive absorption at 300–360 nm, and a minor negative signal at short wavelengths (<250 nm). Control experiments (SI Figure S7) and our recent *operando* UV/Vis studies^[15]^ allowed these features to be assigned to metal‐glucose (300–360) and metal‐methanol (<250 nm) interactions. As these absorptions were common to both samples irrespective of performance, they are not discussed further for brevity. However, a clear difference at longer wavelengths was observed. In the case of Sn‐BEA (Figure 2 A), a positive absorption was immediately formed between 400–480 nm, the intensity of which slowly decreased over the course of the experiment. An excellent inverse correlation between the intensity of this absorption and the selectivity of the catalyst could be made (SI Figure S8), indicating that this absorption relates to by‐product formation. Notably, this signal was absent from the *operando* spectrum of Hf‐BEA, suggesting the absence of various chromophoric by‐products when using this catalyst.

**Figure 3:**
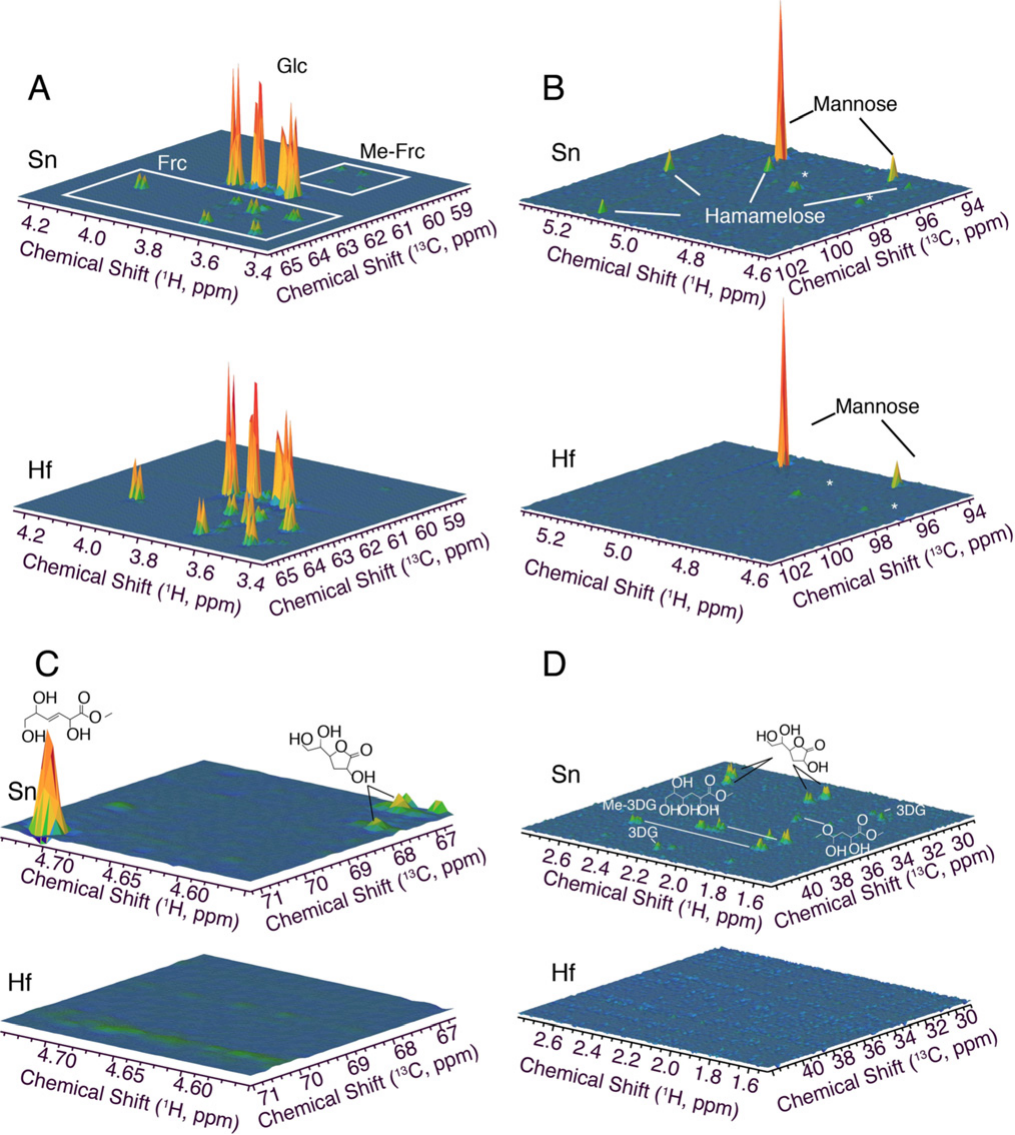
^1^H–^13^C HSQC NMR spectra for NMR analyses of the reaction effluent from Sn‐BEA and Hf‐BEA catalysed GI at 110 °C. Spectra were acquired at 25 °C on an 800 MHz spectrometer equipped with a cryogenically cooled probe. The spectra provide an unbiased representation of the vastly reduced by‐product formation when using Hf‐BEA instead of Sn‐BEA. Various regions of the spectra are expanded in Figures (A)–(D) for clarity.

^1^H‐^13^C HSQC NMR spectroscopy was thus used to obtain an unbiased representation of by‐product formation when using either catalyst. The ^1^H‐^13^C HSQC method was employed as it provided roughly 30‐times higher sensitivity than analogous one‐dimensional ^13^C NMR measurements, whilst providing dramatic improvements in resolution due to its two‐dimensional nature. Moreover, various by‐products in the Lewis acid catalysed conversion of glucose have previously been described using ^1^H‐^13^C HSQC NMR assays, thus facilitating the characterization of post‐reaction solutions.^[16]^ Figure 3 A–D shows the ^1^H‐^13^C HSQC NMR spectra for Sn‐ and Hf‐BEA catalysed GI at 110 °C across various spectral regions. The spectra of both reactions were sampled at different points of time on stream, so that samples of exactly the same level of conversion (20 %) were measured (10 % and 19 % yield of fructose for Sn‐BEA and Hf‐BEA, respectively). As such, spectra were normalised to the quantity of remaining glucose, as verified by NMR standardisation and HPLC‐ELSD. Hence, the volumes of the signals associated to unconverted glucose are identical in both spectra (Figure 3 A).^[16]^ Signals attributed to fructose were much more intense for Hf‐BEA than Sn‐BEA, verifying the improved yield to fructose and hence the improved selectivity of Hf‐BEA. A similar quantity of mannose was also observed during both experiments. However, a number of additional species were observed over Sn‐BEA, none of which were observed for Hf‐BEA. The by‐products observed included methyl fructosides and the branched aldohexose hamamelose, in addition to various glycolytic end products including *trans*‐2,5,6‐trihydroxy‐3‐hexenoic acid methyl ester (THM) and various 3‐deoxy‐γ‐lactones.^[17]^

The diversity of products formed, particularly in the case of Sn‐BEA, indicates that multiple reaction pathways are feasible during GI at these conditions. With the aim of developing structure‐activity relationships to account for these pathways, and to compare the structure and active site speciation of Hf‐BEA to those previously established for Sn‐BEA, spectroscopic studies of Hf‐BEA were performed. Unfortunately, the low gyromagnetic ratio of Hf, its large quadrupole moment and its high‐energy absorption feature (*λ*_max_<200 nm) prohibited its characterisation by classical site‐selective methods.^[18]^ Accordingly, direct and indirect studies were achieved by means of Hf L_3_ edge X‐ray Absorption Spectroscopy (XAS) and chemisorption coupled with FTIR spectroscopy (cIR).

**Figure 4:**
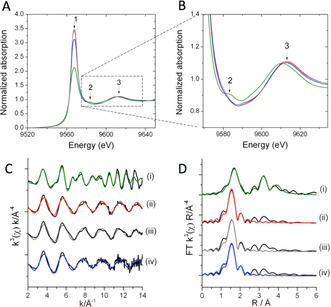
X‐ray absorption spectroscopy data for Hf‐BEA catalysts. A) Hafnium L_3_‐edge XANES of Hf‐BEA catalyst and monoclinic HfO_2_ and B) Expansion of the XANES spectra. Red) fresh catalyst; grey) methanol activated catalyst; blue) used catalyst; green) HfO_2_. 1) White line feature; 2) multiple scattering Hf‐O‐Hf; 3) EXAFS oscillation. C) *k* ^*3*^ weighted *χ* data of Hf‐BEA catalysts and monoclinic HfO_2_. D) Magnitude of Fourier Transform of the L_3_‐edge EXAFS spectra. Samples: i) monoclinic HfO_2_; ii) fresh catalyst; iii) used catalyst; iv) methanol activated catalyst.

Hf L_3_ edge XAS studies were used to gain insight into the Hf active site within the catalyst, before and after reaction, and after MeOH activation (SI Figure S4). Samples were not further treated between operation and measurement, so as to maintain the relevance of the XAS spectra to kinetic performance. Figure 4 A,B shows the X‐ray Absorption Near Edge Structure (XANES) spectra of Hf‐BEA, prior to reaction, after methanol activation, and after 100 h on stream, alongside the spectrum of a monoclinic HfO_2_ standard. The adsorption edges of all catalyst samples, as determined by the maxima of the first derivative of the data, were found to be at 9564.4 eV, compared to 9563.4 eV in monoclinic HfO_2_. The 1 eV shift in adsorption edge of the catalysts compared to HfO_2_ was coupled with a significant increase in the white line feature labelled 1 (2*p* → 5*d* transition) in the catalyst samples, which was greatest in the fresh sample, followed by the methanol activated catalyst and the post reaction material. It is proposed that the formal oxidation state of Hf^IV^ is present in all samples, and that the observed differences indicate a change in the local environment of Hf within the catalyst compared to the monoclinic HfO_2_ standard. Feature 2 was seen in the XANES of HfO_2_, but not in Hf‐BEA at any stage of reaction, and could be attributed to a Hf‐O‐Hf multiple scattering process involving atoms in the second shell. The absence of this feature in the Hf‐BEA catalyst further suggests a different coordination of oxygen to Hf in Hf‐BEA compared to bulk HfO_2_. Feature 3 was the EXAFS oscillation feature of each material.

To complement these findings, Hf L_3_‐edge Extended X‐ray Absorption Fine Structure (EXAFS) studies were performed. The k^x^ weighted data of the samples are shown in Figure 4 C, alongside the corresponding magnitude of the Fourier transform (FT) in Figure 4 D. Based on previous XAS studies of Sn‐BEA,^[20]^ the first feature in the FT data was assigned to Hf‐O scattering paths, while features between 2.5 and 4 Å could be associated to Hf‐Hf scattering and/or second shell Hf‐O paths. Clearly, the first Hf‐O feature in all the catalyst samples (Figure 4 D) was at a shorter distance compared to HfO_2_. Moreover, second shell Hf‐O and/or Hf‐Hf paths were much less significant in the catalysts samples.

Fitting of the EXAFS data (Table 2) revealed two clear first shell Hf‐O paths in the catalyst samples, compared to a single Hf‐O path in monoclinic HfO_2_ that was fitted to represent the 7 very similar Hf‐O paths seen in monoclinic HfO_2_.^[21]^ The first Hf‐O path in all the catalyst samples was found at 2.00 Å, and possessed a coordination number of 4. These values are in excellent agreement to those we determined for Sn‐BEA during previous XAS studies,^[20a]^ and thus this path can be attributed to Hf atoms present in the BEA framework. A second Hf‐O distance, at 2.20 Å with a coordination number of 2, could also be determined in the catalyst samples. Since this second distance is notably longer than the average first shell distance of monoclinic or orthorhombic HfO_2_, it is unlikely to arise from bulk HfO_2_. This interpretation was supported by the poor fit of monoclinic or orthorhombic HfO_2_ models to the second shell paths of all the catalyst samples, alongside the absence of splitting in the oscillation around 9 Å in the *χ* data of all catalysts (Figure 4 C). As such, the second Hf‐O distance could either derive from water bound to the Hf atoms within the framework, or from a small fraction of the Hf being present as HfO_*x*_ clusters. Whilst the absence of HfO_x_ cluster standards makes it impossible to conclusively distinguish between these options, our previous XAS studies of Sn‐BEA materials prior to and following dehydration,^[20a]^ alongside previous studies documenting the hydrated nature of Lewis acidic heteroatoms within the BEA lattice at ambient conditions,^[22]^ lead us to favour the initial hypothesis that is, that the second Hf‐O distance relates to the coordination of two water molecules to the framework Hf species. Thus, we conclude that at least the major fraction of Hf is within the BEA framework, and is present as an isomorphously substituted lattice atom in a hydrated state at ambient conditions. Although this is the first time such XAS studies have been performed for Hf‐BEA catalysts, our findings are supported by previous Pair Distribution Function measurements of Hf‐BEA.^[23]^

**Table 2:** EXAFS fitting data of Hf‐BEA catalysts and monoclinic HfO_2_.^[a]^

Sample	Path	CN	*R* [Å]	2*σ* ^2^ [Å^2^]	*E*_r_ [eV]	*R*‐factor
Mono‐HfO_2_	Hf‐O (1)^[b]^	7^[c]^	2.12(1)	0.011(1)	7(1)	0.007
	Hf‐Hf (1)^[b]^	7^[c]^	3.42(1)	0.008(1)		
	Hf‐O (2)^[b]^	7^[c]^	3.70(3)	0.013(5)		
	Hf‐Hf (2)^[b]^	4^[c]^	4.00(1)	0.005(2)		
Hf‐BEA (fresh)	Hf‐O (1)	4.5(8)	2.00(2)	0.003(2)	7(1)	0.007
	Hf‐O (2)	2.1(5)	2.20(4)	0.004^[d]^		
Hf‐BEA (used)	Hf‐O (1)	4.5(8)	2.00(2)	0.004(2)	8(1)	0.005
	Hf‐O (2)	2.4(5)	2.20(2)	0.004^[d]^		
Hf‐BEA (MeOH activated)	Hf‐O (1)	4.8(8)	2.00(2)	0.004(2)	7(1)	0.007
	Hf‐O (2)	1.9(5)	2.21(5)	0.00^[d]^		

[a] Fitting parameters: 2.2<*k*<12, 1.1<*R*<2.5 (4 for mono‐HfO_2_). Amplitude reduction factor=0.8 (fitted from Mono‐HfO_2_). [b] Multiple path lengths fitted as a single path (i.e Hf‐O(1) comprises of 7 different path lengths). [c] Coordination numbers (CN) fixed to known crystallographic values.^[19]^ [d] 2*σ* ^2^ defined as 1.2 of 2*σ* ^2^ of Hf‐O(1).

The lack of changes in the amplitude of features between 2.5 and 4 Å in the catalyst samples post operation, coupled with the constant coordination numbers for first shell Hf‐O species even after 100 h on stream, strongly suggest that the Hf species present in the catalyst did not change in quantity or structure during reaction, consistent with the excellent stability of the catalyst. Moreover, no changes to the properties of Hf‐BEA were evident after methanol activation. This indicates that the processes occurring during the induction period are too subtle to be probed by XAS, and evidently do not relate to major modifications of the catalyst active sites.

**Figure 5:**
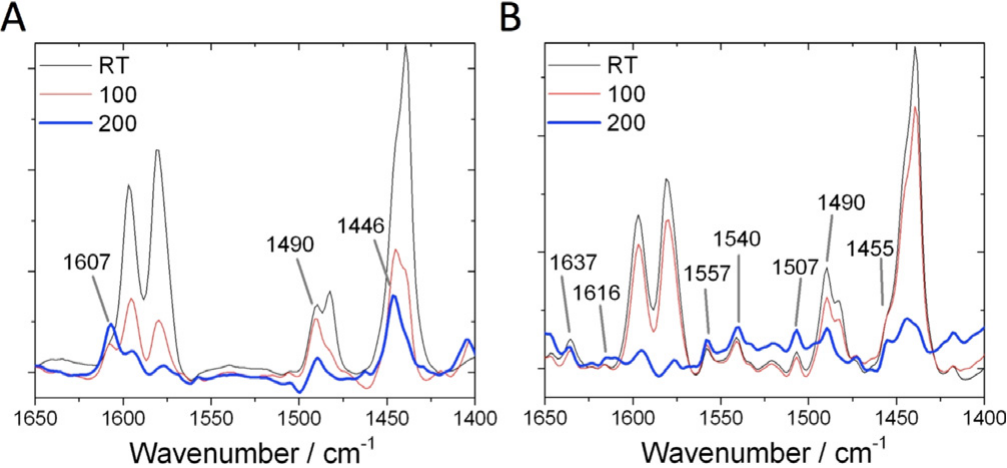
Chemisorption FTIR studies following the interaction of pyridine over A) Hf‐BEA, and B) Sn‐BEA. Spectra are illustrated after outgassing at room temperature (RT), and following thermal treatment at 100–200 °C.

Although it can be concluded that both Sn‐ and Hf‐BEA possess isomorphously substituted Lewis acidic centres, recent studies have indicated that the substitution of different Lewis acids into zeolite lattices can lead to the genesis of materials with very different acidic properties.^[24]^ Thus, to gain insight into the acidic properties of Sn‐ and Hf‐BEA, chemisorption FTIR spectroscopy (cIR) was performed with pyridine as a probe molecule sensitive to both Lewis and Brønsted acidic centres. Figure 5 shows the IR spectra of pyridine adsorbed on (A) Hf‐ and (B) Sn‐BEA in the 1650–1400 cm^−1^ region, following dosing with pyridine vapour at room temperature, and subsequent outgassing up to 200 °C to remove gaseous and weakly bound pyridine, according to the method of Harris et al.^[25a]^ Spectra were background corrected by subtraction of the spectrum of the zeolite catalyst prior to dosing.

Based on previous studies of pyridine adsorption^[25]^ and the behaviour of the vibrations upon thermal treatment, three main classes of vibrations could be assigned; i) vibrations related to physisorbed and/or hydrogen bonded pyridine (1597, 1580, 1482, 1440 cm^−1^); ii) vibrations arising from coordination of pyridine to Lewis acid centres (1620–1600, 1455–1445 cm^−1^), and; iii) vibrations related to pyridine protonated by Brønsted acid centres (1637, 1540 cm^−1^). Since physisorbed and/or hydrogen bonded pyridine vibrations do not relate to the active sites of the catalysts, and vibrations at 1490 cm^−1^ can be generated both by Lewis and Brønsed acid sites, these bands were not followed further.

By interrogation of the cIR profiles (Figure 5 A,B), two major observations could be made. Firstly, although both samples exhibited vibrations related to interaction of pyridine with Lewis acidic centres, the stretching frequencies of these vibrations were present at higher values for Sn‐BEA than Hf‐BEA (1616 vs. 1607 cm^−1^, and 1455 vs. 1446 cm^−1^, respectively). This indicates greater activation of pyridine by the Lewis sites of Sn‐BEA compared to those in Hf‐BEA. Secondly, whilst vibrations associated to the protonation of pyridine were clearly generated by Sn‐BEA (particularly at 1637 and 1540 cm^−1^), such vibrations were totally absent from the spectra of Hf‐BEA. Thus, it can be concluded that Sn‐Beta possesses relatively strong Lewis acid sites alongside Brønsted acidic active sites, whereas Hf‐BEA only contains Lewis acid active sites, which are of a lower strength to those found in Sn‐BEA. We note that the observation of Brønsted acidity in Sn‐BEA is supported by recent NMR studies,^[24a]^ whilst the weaker Lewis acid strength of Hf‐BEA relative to Sn‐BEA is supported by ^15^N MAS NMR studies of various Lewis and Brønsted acidic silicates.^[26]^

We hypothesise that these differences in acidic nature are responsible for the excellent performance of Hf‐BEA; Whilst the stronger Lewis and Brønsted acidic sites in Sn‐BEA result in a catalyst that is initially more active than Hf‐BEA in terms of glucose conversion (see TOF in Table 1 and conversion in Figure 1), increased conversion is achieved due to initiation of several competing processes over multiple active sites that co‐exist in the catalyst. In contrast, the monofunctional Lewis acidity in Hf‐BEA results in isomerisation being achieved at unprecedentedly high selectivity (>90 %).

**Figure 6:**
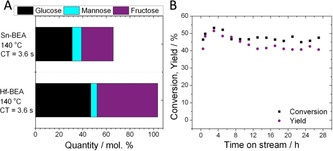
Kinetic data for the isomerisation of glucose to fructose over Sn‐ and Hf‐containing BEA zeolites at 140 °C. A) Distribution of products attained over Sn‐BEA and Hf‐BEA at maximal conversion at 140 °C. B) Time on stream analysis of Hf‐BEA at 140 °C over a 28 h period. General reaction conditions: 1 wt % glucose in methanol, 140 °C. Precise conditions including WHSV of each experiment are provided in Supporting Information Table S1.

Given that higher reaction temperatures favour thermodynamic equilibrium yields^[7b]^ and boost reactor productivity (space‐time‐yield), the activity of Hf‐BEA for intensified GI at elevated temperature (140 °C) was explored. As can be seen, the excellent performance of Hf‐BEA was maintained even at 140 °C, with fructose still produced at yields of up to 50 % at a selectivity 94 %, but with a three‐fold increase in space‐time‐yield over that achieved at 110 °C (4.12 g (Fru) cm^−3^ h^−1^ versus 1.3 g (Fru) cm^−3^ h^−1^) (Figure 6 A). In contrast, poor fructose yield, selectivity and carbon balance were observed with Sn‐BEA at 140 °C (2.1 g (Fru) cm^−3^ h^−1^). In addition, excellent stability for Hf‐BEA was observed over a 28 h period of operation (Figure 6 B). Notably, the induction period observed at 110 °C was also eliminated at higher temperature, resulting in a catalytic process that was highly productive and selective from the very first moments of operation without requirement of a preceding methanol activation step (SI Figure S4). Furthermore, high levels of productivity were maintained even in the presence of additional water (SI Figure S9). Notably, separation of the product mixture by reduced pressure distillation also revealed that the isolated product formed at 140 °C was a white, crystalline solid, confirming the absence of Maillard browning processes even during high temperature operation with Hf‐BEA (SI Figure S10).

## Conclusion

Hf‐containing zeolites are shown to be unique catalysts for glucose‐fructose isomersation, enabling unprecedented single pass yields of 58 % to be achieved at a fructose selectivity of 94 %, for over 100 hours on stream. Spectroscopic studies by XAS, chemisorption FTIR, *operando* UV/Vis and ^1^H‐^13^C HSQC NMR suggest the activity of Hf‐BEA arises from the presence of isolated Hf^IV^ atoms with monofunctional Lewis acidic properties. Although catalytic activity is only initially observed after a period of activation in the reactor, this can be bypassed by pre‐treating Hf‐BEA in the reaction solvent, or by operating the reaction at higher temperature. Optimisation of the system permits selective glucose‐fructose isomerisation to be achieved even during intensified continuous operation at 140 °C, which no chemical or biological catalyst has yet been able to achieve. The reasons behind the induction period observed for Hf‐BEA at lower temperature, and its higher stability against deactivation compared to other Lewis acidic zeolites, remain the focus of our on‐going investigations.

## Conflict of interest

The authors declare no conflict of interest.

## Supporting information

As a service to our authors and readers, this journal provides supporting information supplied by the authors. Such materials are peer reviewed and may be re‐organized for online delivery, but are not copy‐edited or typeset. Technical support issues arising from supporting information (other than missing files) should be addressed to the authors.

Supplementary
