# Supplementary material for: Solvent‐Activated Hafnium‐Containing Zeolites Enable Selective and Continuous Glucose–Fructose Isomerisation
Source: Angew Chem Int Ed Engl. 2020 Aug 31;59(45):20017–23. doi: 10.1002/anie.202006718 (PMC7818259; doi:10.1002/anie.202006718)
Supplement: Supplementary file 1 — Supplementary [file ANIE-59-20017-s001.pdf]

Angewandte Chemie
Eine Zeitschrift der Gesellschaft Deutscher Chemiker

Supporting Information

# Solvent-Activated Hafnium-Containing Zeolites Enable Selective and Continuous Glucose–Fructose Isomerisation

*Luca Botti, Simon A. Kondrat, Ricardo Navar, Daniele Padovan, Juan S. Martinez-Espin, Sebastian Meier, and Ceri Hammond**

anie_202006718_sm_miscellaneous_information.pdf

# Supporting Information

---

Table of Contents

Catalyst synthesis procedure...…..…………..……………………….………………….….… 2
Details of kinetic studies.…………………………………………………….………….....…..….. 2-3
Details of catalyst characterisation……………………………………….…………………...3-4
Figures…………………………………………………………………….…….……….…….....5-10
Tables………………………………………………………………………..…………….....…...11

**Catalyst synthesis**

The hydrothermal synthesis of metal incorporated BEA zeolites was performed according to the following protocol: 30.6 g of tetraethyl orthosilicate (TEOS, Sigma Aldrich, 98%) was added to 33.1 g of tetraethylammonium hydroxide (TEAOH, Sigma Aldrich, 35%) under careful stirring, resulting in the formation of a two-phase mixture. After 60-90 min, a single phase was obtained, and the desired amount of the metal source ($SnCl_4{\cdot}5H_2O$ or $HfCl_4$ or $ZrCl_4$, Sigma Aldrich > 99.5%) dissolved in 2.0 mL of $H_2O$ was added dropwise. The solution was then left for several hours under stirring until a viscous gel was formed. Formation of the final solid gel was achieved by addition of 3.1 g of hydrofluoric acid (HF, Fischer Chemicals, 50%) in 1.6 g of demineralized $H_2O$. The molar composition of the final gel was; 1.0Si: 0.005Sn: 0.02$Cl^-$: 0.55$TEA^+$: 0.55$F^-$: 7.5$H_2O$. The obtained gel was transferred to a Teflon lined stainless steel autoclave, and heated at 140 °C for a total 7 days. The crystals obtained were filtered and washed with deionised water. Removal of the organic template was achieved by calcination at 550 °C (2 °C $min^{-1}$) for 6 h under static air.

**Kinetic and analytical studies**

Continuous glucose isomerisation reactions were performed in a plug flow, stainless steel tubular reactor. The catalyst was pelletised (size fraction 63-77 μm) and densely packed into a ¼" stainless steel tube (4.1 mm internal diameter). Two plugs of quartz wool, and a frit of 0.5 μm, held the catalyst in place. The reactor was heated by immersion in a thermostatted oil bath, and pressurization was achieved by means of a backpressure regulator. Aliquots of the reaction solution were taken periodically from a sampling valve placed after the reactor. Samples were analysed by means of an Agilent 1260 Infinity HPLC, equipped with a Hi-Plex-Ca column and ELS detector. Quantification was achieved by reference against an external standard (sorbitol), which was added to the sample prior to injection into the HPLC. The conditions of each catalytic experiment are provided in ESI Table S1.

High field liquid NMR analysis on the samples was performed on a Bruker Avance III 800 MHz spectrometer, equipped with a TCI cryoprobe at 25 °C. The samples were dried under a flow of nitrogen at 25°C, and subsequently re-diluted in deuterated methanol. $^{1}H$-$^{13}C$ HSQC spectra were

acquired by sampling the FID in the $^{1}H$ and $^{13}C$ dimensions by 1024 and 512 complex data points, respectively, during acquisition times of 142 milliseconds ($^{1}H$) and 18 milliseconds ($^{13}C$). All spectra were processed with ample zero filling in both dimensions using Bruker Topspin 3.5 pl6.

Batch glucose studies were performed in a pressurised ACE tubular glass reactor, heated by immersion in an oil bath. The reactor was filled with 4 g of reactant solution (1 wt. % glucose in methanol) and the appropriate amount of catalyst required to achieve a glucose/metal molar ratio of 50. Samples were periodically collected and analysed by HPLC as described above.

**Catalyst Characterisation**

XRD patterns were acquired using a PANalytical X'Pert PRO X-ray diffractometer. A CuKα radiation source (40 kV and 30 mA) was utilised. Diffraction patterns were recorded between 6–55° 2θ (step size 0.0167°, time/step = 150 s, total time = 1 h). Specific surface area was determined from nitrogen adsorption using the BET equation, and microporous volume was determined from nitrogen adsorption isotherms using the t-plot method. Porosimetry and surface area measurements were both performed on a Quantachrome Autosorb-iQ-MP/XR, and samples were degassed prior to use (115 °C, 6 h, nitrogen flow). Pyridine adsorption was monitored by DRIFT measurements, achieved by use of a Bruker Tensor spectrometer equipped with a Harrick praying mantis cell. Spectra were recorded over a range of 4000-650 $cm^{-1}$, at a resolution of 2 $cm^{-1}$. Prior to dosing with pyridine, the zeolite powder was heated in a flow of nitrogen (100 °C, 40 mL $min^{-1}$, 0.5 h). Pyridine was dosed onto the sample by redirecting the gas stream through a saturator module containing pyridine. Samples were maintained at 25 °C during adsorption of pyridine for 10 minutes. Afterwards the pyridine saturator was disconnected from the cell, and the temperature was increased at a rate of 5 °C $min^{-1}$ up to 200 °C whilst maintaining the flow of nitrogen. Operando UV−Vis measurements were performed with a homemade tubular reactor equipped with a fibre optic UV−Vis probe. UV−Vis measurements were performed by use of a light source (Ocean Optics DH-2000), spectrometer (Maya 2000 Pro, Ocean Optics), and a 600-μm UV−vis fibre. The light was directed onto an optically transparent reactor column, located within a heated aluminum block.

X-ray absorption spectra were collected on B18, Diamond Light Source at the Harwell campus, UK. Samples were analysed as pellets in transmission mode at room temperature using the fast-scanning Si(111) double crystal monochromator. Data processing, including alignment, normalisation and background removal, was performed using the Demeter software package (Athena). Analysis of the EXAFS data was performed using IFEFFIT within the Artimus software package (Ravel, Journal of Synchrotron Radiation, 2005, 12, 537 and Newville, Journal of Synchrotron Radiation, 2001, 8, 322). $1^{st}$ shell path lengths were fitted at all k weighted $\chi$ data using a k space window of $2.2 < k < 12$, $1.1 < R < 2.5$. The R range was extended to 4 when fitting $2^{nd}$ shells. A standard of monoclinic $HfO_2$ was fitted using simplified paths of single paths for Hr-O first shell, Hr-Hr and second shell Hr-O distances as reported by Erenburg and co-workers. Successfully fitted second shell paths were applied to the fitting of catalyst EXAFS data, with path lengths and coordination numbers being refined. However no acceptable results were obtained when applying such second shell paths to the catalyst spectra.

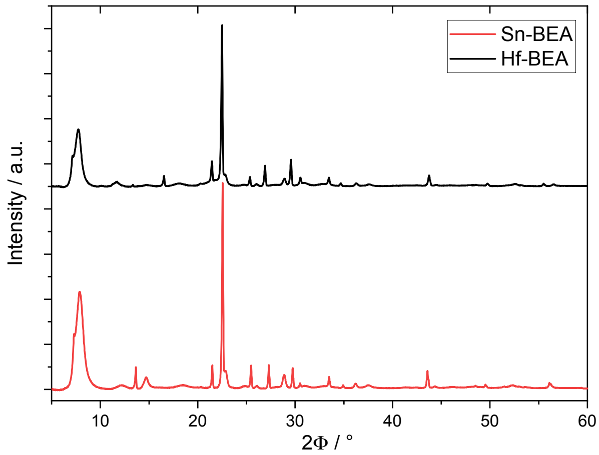


**Figure S1.** X-Ray diffraction patterns for Sn-BEA and Hf-BEA catalysts prepared by hydrothermal synthesis.

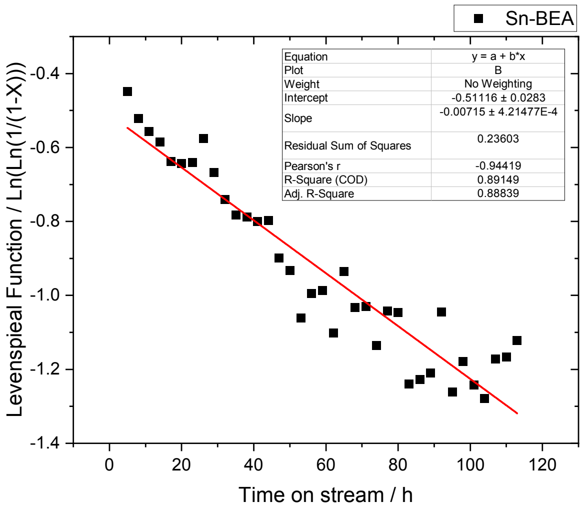


**Figure S2.** Levenspiel plot for the determination of the deactivation constant ($k_d$) of Sn-BEA during the continuous isomerisation of glucose to fructose. Experimental conditions are described in Table S1, Entry 1.

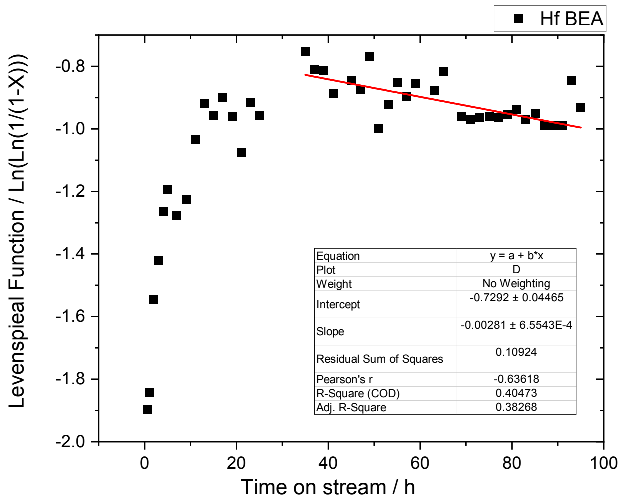


**Figure S3.** Levenspiel plot for the determination of the deactivation constant ($k_d$) of Hf-BEA during the continuous isomerisation of glucose to fructose. Experimental conditions are described in Table S1, Entry 2

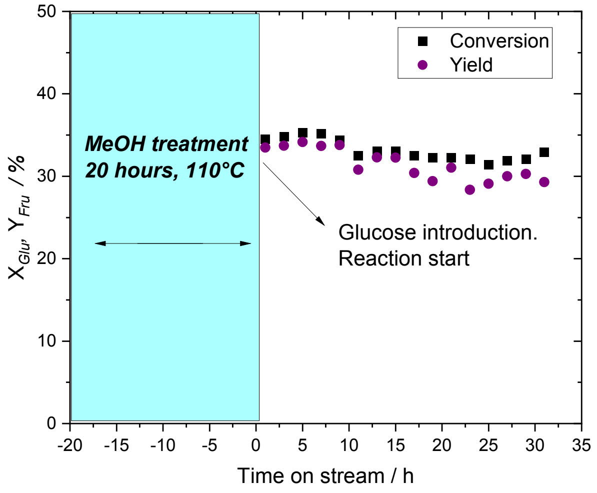


**Figure S4.** Catalytic performance of Hf-BEA for continuous glucose isomerisation following treatment in methanol flow for 20 h at 110 °C prior to introduction of glucose into the feed. The conditions for the operational phase are reported in Table S1, Entry 2. Methanol treatment was performed at the same flow rate as used in the reaction.

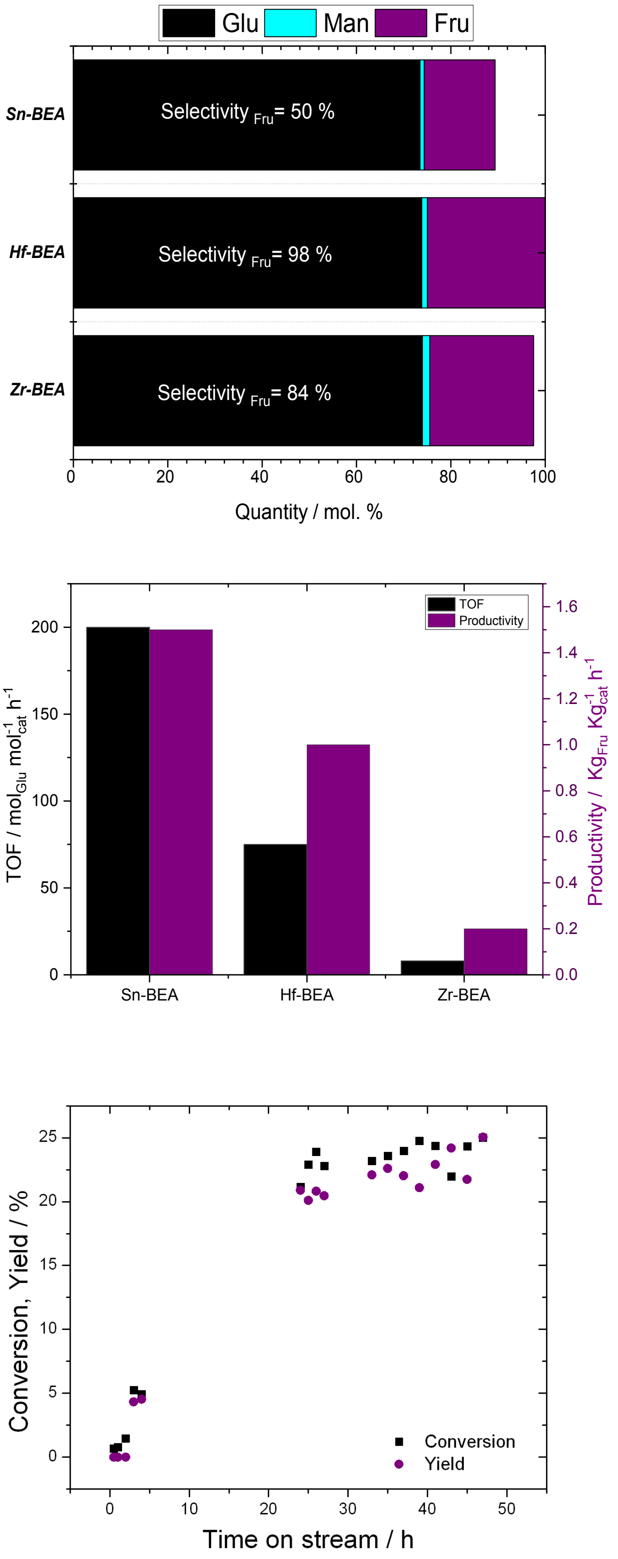


**Figure S5.** (Top) Product distribution achieved during glucose isomerisation over Sn-BEA, Hf-BEA and Zr-BEA at the same level of conversion (25 %). (Middle) TOF and productivity value for Sn-BEA, Hf-BEA and Zr-BEA. (Bottom) Time on stream data for Zr-BEA, illustrating its induction period. Experimental conditions are reported in Table S1, Entries 1-3. The TOF and productivity data were measured at maximal conversion, whereas the product distribution is demonstrated at an identical level of conversion (25 %) for more rigorous comparison of selectivity.

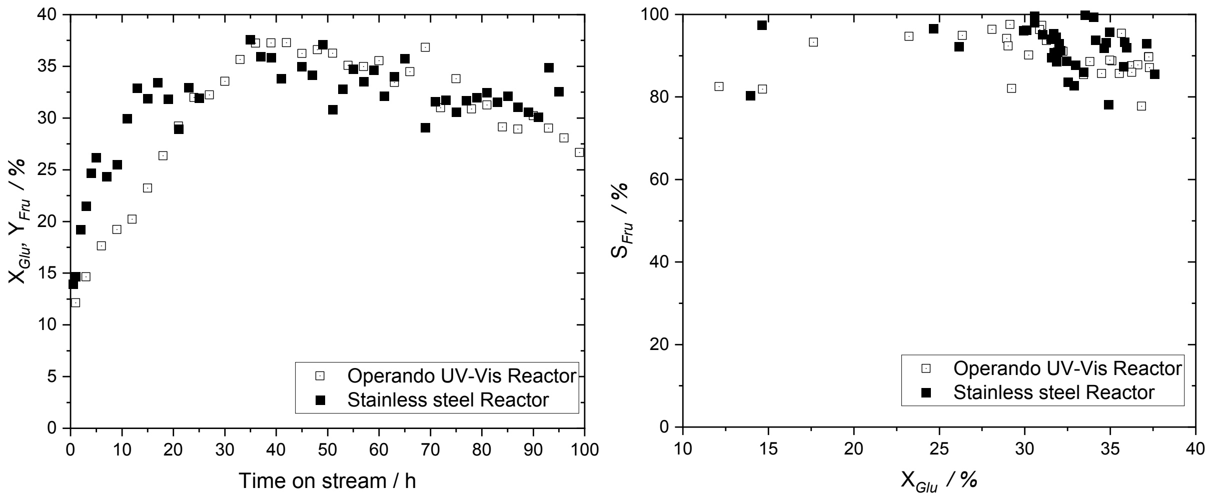


**Figure S6.** Kinetic activity of Hf-BEA for glucose isomerisation when the reaction was performed in a conventional stainless steel reactor (black squares) and the *operando* UV-Vis reactor (open squares). Experiment conditions are shown in Table S1, Entry 3.

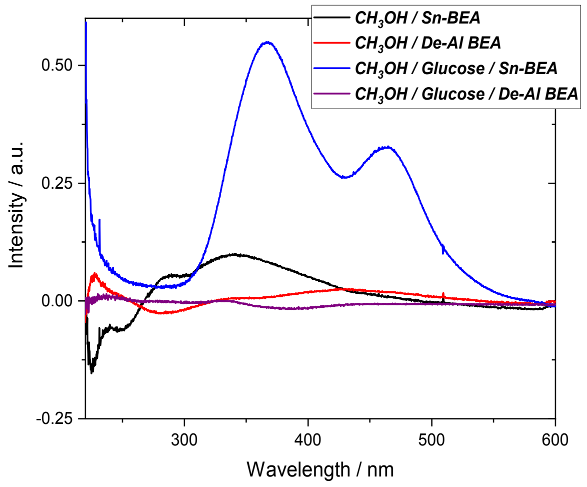


**Figure S7.** Control experiments performed in the *operando* UV-Vis reactor for different catalysts and reaction components.

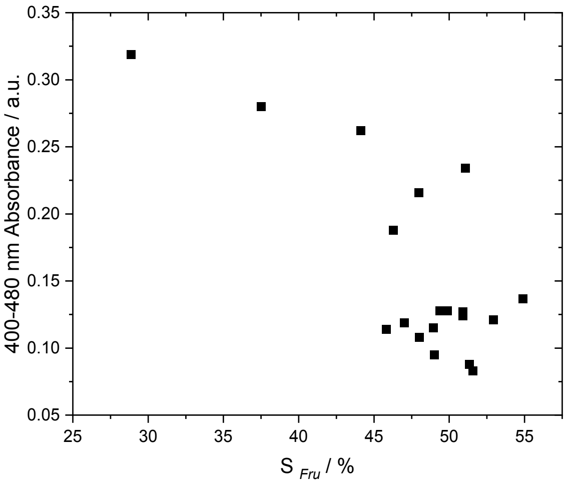


**Figure S8.** Correlation between the absorption observed between 400-480 nm in the spectrum of Sn-BEA catalysed glucose isomerisation and the selectivity to fructose exhibited by the catalyst.

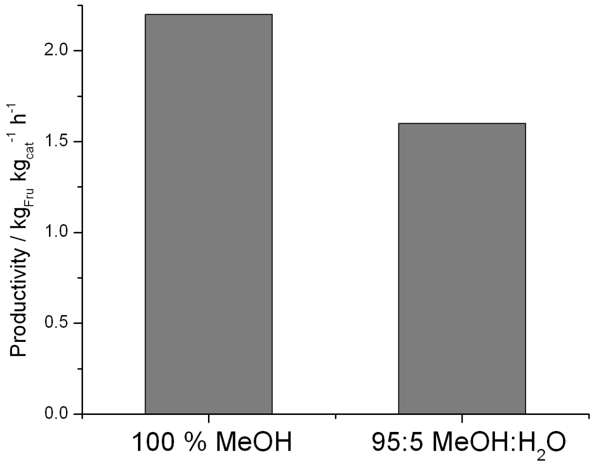


**Figure S9.** Productivity of Hf-BEA for the isomerisation of glucose to fructose at 140 °C when the solvent is methanol (MeOH) and a mixture of water/methanol (95:5 w/w, MeOH:$H_2O$). Asides from the choice of solvent, the experimental conditions of both tests were identical and are described in Table S1, Entry 4.

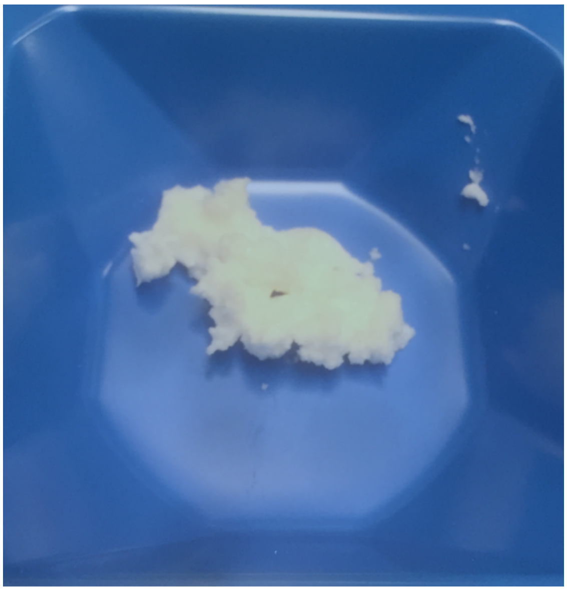

**Figure S10.** Colour of the sugar product purified from the isomerisation reaction catalysed by Hf-BEA zeolite at 140 °C with 50% conversion and 94% selectivity. Experimental conditions are given in Table S1, Entry 4.

**Table S1.** Experimental conditions for the continuous GI reaction showed along the manuscript.

| Entry | Catalyst | Reaction feed | Temp. (°C) | Flow (mL/min) | Mass. Catalyst (g) | WHSV* (Kg $Kg^{-1}$ $h^{-1}$) | C.T. (τ)**(s) |
|---|---|---|---|---|---|---|---|
| 1 | Hf-BEA | 1 wt. % glucose in methanol | 110 | 0.65 | 0.1 | 3.1 | 8.4 |
| 2 | Sn-BEA | 1 wt. % glucose in methanol | 110 | 1.5 | 0.1 | 7.2 | 3.6 |
| 3 | Zr-BEA | 1 wt. % glucose in methanol | 110 | 0.1 | 0.1 | 0.48 | 54 |
| 4 | Hf-BEA | 1 wt. % glucose in methanol | 140 | 1.5 | 0.1 | 7.2 | 3.6 |
| 5 | Sn-BEA | 1 wt. % glucose in methanol | 140 | 1.5 | 0.1 | 7.2 | 3.6 |

*Weight Hourly Space Velocity: mass of substrate flowed per hour over 1 kg of catalyst
**Contact time: time of interaction between the substrate and the catalyst
